# Supplementary material for: A Critical View of the Application of the APEX Software (Aqueous Photochemistry of Environmentally-Occurring Xenobiotics) to Predict Photoreaction Kinetics in Surface Freshwaters
Source: Molecules. 2019 Dec 18;25(1):9. doi: 10.3390/molecules25010009 (PMC7017383; doi:10.3390/molecules25010009)
Supplement: Supplementary file 1 [file molecules-25-00009-s001.zip › 3.2.4_gcc-4.4.0.zip!/3.2.4_gcc-4.4.0/3.2.4_gcc-4.4.0/doc/octave/html/interpreter/Element_002dby_002delement-Boolean-Operators.html]

Element-by-element Boolean Operators - Untitled


Next: Short-circuit Boolean Operators,
Up: Boolean Expressions


---

#### 8.5.1 Element-by-element Boolean Operators

An element-by-element boolean expression is a combination of
comparison expressions using the boolean
operators “or” (|), “and” (&), and “not” (!),
along with parentheses to control nesting. The truth of the boolean
expression is computed by combining the truth values of the
corresponding elements of the component expressions. A value is
considered to be false if it is zero, and true otherwise.

Element-by-element boolean expressions can be used wherever comparison
expressions can be used. They can be used in `if` and `while`
statements. However, a matrix value used as the condition in an
`if` or `while` statement is only true if *all* of its
elements are nonzero.

Like comparison operations, each element of an element-by-element
boolean expression also has a numeric value (1 if true, 0 if false) that
comes into play if the result of the boolean expression is stored in a
variable, or used in arithmetic.

Here are descriptions of the three element-by-element boolean operators.

boolean1 `&` boolean2: Elements of the result are true if both corresponding elements of boolean1 and boolean2 are true. boolean1 `|` boolean2: Elements of the result are true if either of the corresponding elements of boolean1 or boolean2 is true. `!` boolean `~` boolean: Each element of the result is true if the corresponding element of boolean is false.

For matrix operands, these operators work on an element-by-element
basis. For example, the expression

```
     [1, 0; 0, 1] & [1, 0; 2, 3]
```

returns a two by two identity matrix.

For the binary operators, the dimensions of the operands must conform if
both are matrices. If one of the operands is a scalar and the other a
matrix, the operator is applied to the scalar and each element of the
matrix.

For the binary element-by-element boolean operators, both subexpressions
boolean1 and boolean2 are evaluated before computing the
result. This can make a difference when the expressions have side
effects. For example, in the expression

```
     a & b++
```

the value of the variable b is incremented even if the variable
a is zero.

This behavior is necessary for the boolean operators to work as
described for matrix-valued operands.
